# Supplementary material for: Active site specificity profiling datasets of matrix metalloproteinases (MMPs) 1, 2, 3, 7, 8, 9, 12, 13 and 14
Source: Data Brief. 2016 Feb 22;7:299–310. doi: 10.1016/j.dib.2016.02.036 (PMC4777984; doi:10.1016/j.dib.2016.02.036)
Supplement: Supplementary file 10 — Supplementary material [file mmc10.zip › WebPICS_hMMP7_T_1%/P2.html]

 

PICS results


|  |  |
| --- | --- |
| **P2\_A**  37 in 291 sites   12.7 %    effects > 10 perc. pnts.  (vice-versa in brackets)  P1\_E: 11.6 (11.6) |  |
| **P2\_C**  7 in 291 sites   2.4 %    effects > 10 perc. pnts.  (vice-versa in brackets)  P3prime\_N: 52.6 (28.4) |  |
| **P2\_D**  25 in 291 sites   8.6 %    effects > 10 perc. pnts.  (vice-versa in brackets)  P1\_F: 10.5 (16.4) |  |
| **P2\_E**  32 in 291 sites   11.0 %    effects > 10 perc. pnts.  (vice-versa in brackets)  P1\_D: 13.0 (15.9)   P1prime\_M: 14.3 (35.2)   P2prime\_I: 10.6 (10.2) |  |
| **P2\_G**  32 in 291 sites   11.0 %    effects > 10 perc. pnts.  (vice-versa in brackets)  P1\_G: 19.5 (25.0)   P3prime\_G: 12.6 (11.2) |  |
| **P2\_M**  10 in 291 sites   3.4 %    effects > 10 perc. pnts.  (vice-versa in brackets)  P2prime\_M: 34.5 (21.6)   P3prime\_N: 25.5 (19.7) |  |
| **P2\_N**  23 in 291 sites   7.9 %    effects > 10 perc. pnts.  (vice-versa in brackets)  P3prime\_I: 10.9 (13.2) |  |
| **P2\_Q**  20 in 291 sites   6.9 %    effects > 10 perc. pnts.  (vice-versa in brackets)  P2prime\_H: 11.9 (26.4)   P3prime\_D: 12.4 (11.3)   P3prime\_Q: 13.1 (13.1) |  |
| **P2\_Y**  10 in 291 sites   3.4 %    effects > 10 perc. pnts.  (vice-versa in brackets)  P3\_E: 23.1 (11.6)   P1\_N: 38.7 (11.8)   P3prime\_F: 15.2 (10.9) |  |
